# Persistent mammalian orthoreovirus, coxsackievirus and adenovirus co-infection in a child with a primary immunodeficiency detected by metagenomic sequencing: a case report

**DOI:** 10.1186/s12879-018-2946-7

**Published:** 2018-01-11

**Authors:** Dagmara W. Lewandowska, Riccarda Capaul, Seraina Prader, Osvaldo Zagordi, Fabienne-Desirée Geissberger, Martin Kügler, Marcus Knorr, Christoph Berger, Tayfun Güngör, Janine Reichenbach, Cyril Shah, Jürg Böni, Andrea Zbinden, Alexandra Trkola, Jana Pachlopnik Schmid, Michael Huber

**Affiliations:** 1 Institute of Medical Virology, University of Zurich, Winterthurerstrasse 190, 8057 Zurich, Switzerland; 2 Division of Immunology, University Children’s Hospital Zurich, Steinwiesstrasse 75, 8032 Zurich, Switzerland; 3 Division of Infectious Diseases and Hospital Epidemiology, University Children’s Hospital Zurich, Steinwiesstrasse 75, 8032 Zurich, Switzerland; 4 Division of Stem Cell Transplantation, University Children’s Hospital Zurich, Rämistrasse 100, 8091 Zurich, Switzerland; 5 Present address: Unilabs, Ringstrasse 12, 8600 Dübendorf, Switzerland

**Keywords:** Orthoreovirus, Coxsackievirus, Adenovirus, Primary immunodeficiency, Metagenomic sequencing

## Abstract

**Background:**

We report a rare case of *Mammalian orthoreovirus* (MRV) infection in a child with a primary immunodeficiency (PID). Infections with Mammalian orthoreovirus are very rare and probably of zoonotic origin. Only a few cases have been described so far, including one with similar pathogenesis as in our case.

**Case presentation:**

The patient, age 11, presented with flu-like symptoms and persistent severe diarrhea. *Enterovirus* has been detected over several months, however, exact typing of a positive cell culture remained inconclusive. Unbiased metagenomic sequencing then detected MRV in stool samples from several time points. The sequencing approach further revealed co-infection with a recombinant *Coxsackievirus* and *Adenovirus*. MRV-specific antibodies detected by immunofluorescence proved that the patient seroconverted.

**Conclusion:**

This case highlights the potential of unbiased metagenomic sequencing in supplementing routine diagnostic methods, especially in situations of chronic infection with multiple viruses as seen here in an immunocompromised host. The origin, transmission routes and implications of MRV infection in humans merit further investigation.

**Electronic supplementary material:**

The online version of this article (10.1186/s12879-018-2946-7) contains supplementary material, which is available to authorized users.

## Background

Individuals suffering from primary immunodeficiencies (PIDs) are prone to a variety of infections, and some types of PIDs can predispose the affected individuals to particular pathogens. Infections that are usually controlled and asymptomatic in immunocompetent individuals often cause chronic, active disease in immunocompromised individuals [1].

Rapid diagnosis of viral infections is crucial in immunocompromised patients. While a range of established molecular tests can detect specific viruses, high-throughput metagenomic sequencing is based on virus-sequence independent amplification of nucleic acids isolated directly from clinical samples; as such, it has the potential to identify any virus in an “open diagnostics” approach [2, 3]. Hence, this approach can detect rare viruses that are not included in routine diagnostic panels and viruses with sequence variations that would otherwise be missed [4]. Here we used metagenomic sequencing to complement routine diagnostic methods in a child with a PID suffering from persistent diarrhea.

## Case presentation

We report on a female child living in Switzerland with a combined B- and T-cell immunodeficiency, hypogammaglobulinaemia and autoimmunity (diabetes mellitus) under immunoglobulin replacement therapy. In early February 2014, the patient, aged 11 at that time, presented with flu-like symptoms with cough, headache, fever and severe diarrhea. Although the other symptoms resolved, the diarrhea persisted. In view of low initial calprotectin levels (100–400 μg/g), a fecal marker for intestinal inflammation, the diarrhea was considered as caused by the underlying PID and not by an infectious agent. However, a rise in calprotectin level to 4000 μg/g in March 2015 prompted us to initiate virological investigations. *Enterovirus* had been detected over several months using a specific RT-PCR (Additional file 1: Table S1) and was thus considered as causative agent although other viruses were not tested for during this time period.

As symptoms continued, additional testing was performed. A stool sample (November 3rd 2015) was positive in Caco-2 cell culture showing a cytopathic effect (CPE) after 9 days of incubation. The culture sample stained positive with a pool of anti-enterovirus monoclonal antibodies, but we did not succeed to further subtype the suspected enterovirus using routine immunostaining methods. In order to identify the virus amplified in the Caco-2 cell culture, we applied unbiased metagenomic sequencing as previously described [4]. For three sampling time points in September and November 2015, we sequenced both the Caco-2 culture supernatant and the original stool suspension. We detected many reads of *Mammalian orthoreovirus 3* (MRV-3) in the cell culture supernatant and reads of *Coxsackievirus A* (CV-A) in the original stool suspension (Table 1). No virus reads were detected in the supernatant of a non-infected Caco-2 cell culture used as a negative control.

**Table 1:** Retrospective viral diagnostics using cell culture, metagenomic sequencing and specific PCRs

		Cell culture (CPE)	Metagenomic sequencing (number of reads)		Specific PCR (threshold cycle)				
Date	Sample	Original material	Original material	Cell culture supernatant	MRV-3 original material	MRV-3 cell culture supernatant	CV-A original material	HAdV original material	HAdV cell culture supernatant
25.09.2014	colon biopsy	na	na	na	undet	na	37.3	undet	na
02.01.2015	stool	negative	CV-A (9′618)	na	undet	na	20.1	33.0	na
29.04.2015	colon biopsy	na	na	na	undet	na	undet	undet	na
30.04.2015	stool	positive	CV-A (555)	HAdV-C (328′173)	undet	undet	23.6	35.3	12.0
26.05.2015	stool	positive	CV-A (746′862) HAdV-C (6′131)	HAdV-C (228′718)	undet	undet	28.0	40.5	13.8
30.06.2015	stool	positive	na	HAdV-C (439′345)	undet	undet	24.0	37.0	14.6
14.07.2015	stool	positive	na	HAdV-C (452′131)	undet	undet	23.5	33.3	12.2
10.09.2015	stool	positive	CV-A (195′249)	MRV-3 (6′580)	undet	21.6	20.1	undet	na
3.11.2015	stool	positive	CV-A (414)	MRV-3 (2′742)	35.8	22.2	27.3	undet	na
25.11.2015	stool	positive	CV-A (1′549’977) HAdV-C (102)	MRV-3 (850)	37.3	24.0	21.2	undet	undet
09.02.2016	stool	na	na	na	undet	na	14.5	undet	na
18.03.2016	stool	negative	na	na	undet	na	undet	undet	na
04.05.2016	stool	negative	na	na	undet	na	23.5	38.24	na

*na* not done or not available, *undet* undetermined threshold cycle (> 45), cell culture (CPE) was tested on Caco-2 cells

To trace the timing of these two virus infections we conducted a retrospective analysis of the available stool samples by both cell culture and metagenomic sequencing (Table 1). In cell cultures, a CPE was visible after about 14 days of culturing. By sequencing the cell culture supernatants, we found numerous reads for *Human adenovirus C* (HAdV-C) in these earlier time points, but none for MRV-3. By sequencing original stool suspensions, we identified reads corresponding to the previously identified CV-A.

We verified all the metagenomics analyses by specific PCRs designed for the isolates in this study (Table 1). In addition to confirming MRV-3 in the three cell cultures supernatants with MRV-3 reads, we also detected MRV-3 in two corresponding stool suspensions by specific PCR. CV-A was confirmed in all stool suspensions and in one of two colon biopsies; HAdV-C was confirmed in all tested cell cultures and stool suspensions (Table 1).

**Fig. 1:**
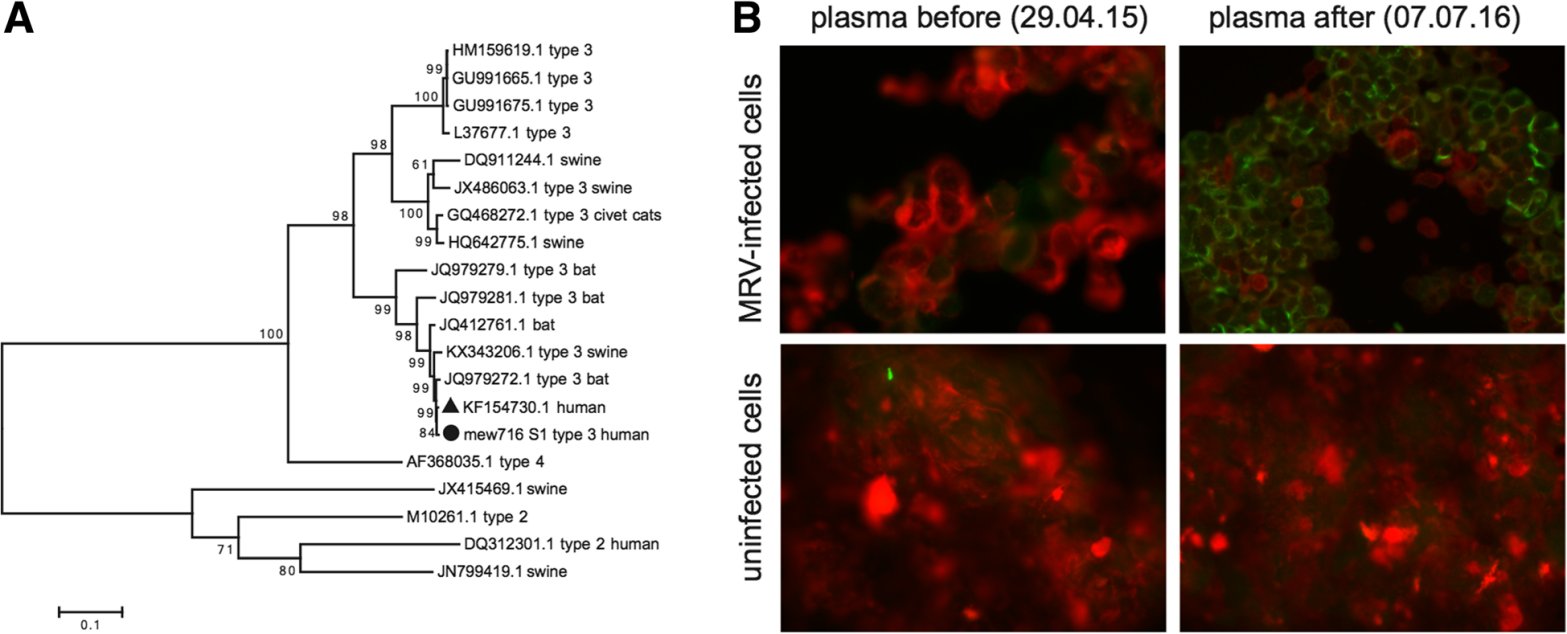
Mammalian orthoreovirus infection confirmed by phylogenetic analysis and immunofluorescence staining. **a** Phylogenetic analysis of Mammalian orthoreovirus segment S1 isolated in this study (circle, mew716_S1_type_3_human) reveals a close relationship with previously described isolates identified in a child in Slovenia (triangle, KF154730), bats in Germany (JQ412761), and pigs in Italy (KX343206). Phylogenetic trees were constructed in MEGA7 using the Maximum Likelihood method based on the Kimura 2-parameter model. Bootstrap values from 1000 tries are shown. MRV-type and host species are depicted if available. **b** Anti-MRV-3 immunofluorescence of patient plasma before and after seroconversion on MRV-3-infected and uninfected Caco-2 cells

To define the MRV-3 infection in more detail, we combined sequencing reads from all time points and reconstructed a consensus sequence that covered the full coding sequences in all 10 segments of the MRV-3 isolate (GenBank KX932029 - KX932038). Phylogenetic analyses showed that the MRV-3 sequence isolated in this study was most related in all 10 segments to an isolate from a child in Slovenia [5] and further clustered with isolates from bats in Germany [6] and pigs in Italy [7] (Fig. 1a and Additional file 1: Figures S1-S9).

In order to proof active replication and diagnosis of MRV infection, we performed immunofluorescence for the detection of MRV-specific antibodies of the patient. Patient plasma from time points after MRV detection were positive for anti-MRV IgG antibodies showing that the patient seroconverted (Fig. 1b).

We were able to reconstruct the full-length coxsackievirus genome present in this patient using additional sequence information from sequencing with CV-A22 serotype consensus primers. Genotyping and bootscan analysis revealed that the isolate (GenBank KX932039) probably resulted from recombination between CV-A19 and CV-A22 (Additional file 1: Figures S10 and S11). The detected HAdV-C was type 2, strain human/ARG/A15932/2002/2[P2H2F2] (JX173079.1).

## Discussion and conclusions

Using a metagenomic sequencing approach, we detected multiple virus infections in a child with PID and persistant diarrhea. Although routine PCR methods detected *Enterovirus* in stool samples for a prolonged period of time, an attempt to subtype the virus after cell culturing was not successful. In order to resolve this, we performed metagenomic sequencing of cell culture supernatants and stool suspensions.

The most unexpected finding was the infection with MRV-3, proven by metagenomic sequencing and serology of samples from several time points. MRVs belong to the *Reoviridae* family, a group of non-enveloped dsRNA viruses with 10 genome segments and have been isolated from a wide range of mammalian hosts and in a variety of clinical contexts [5–7]. To date, the diseases associated with MRV infections in humans include respiratory disease [8], meningitis [9–11], acute necrotizing encephalopathy [12] and (in a similar case involving a child living in Slovenia) acute gastroenteritis [5]. Zoonotic transmission is often suspected [6, 13, 14]. Our patient lives close to a farm and might have come in contact with pets, farm animals and animal feces making a zoonotic infection conceivable, however, due to the lack of data on MRV distribution and appropriate samples we can only hypothesize on the transmission route [7]. Stool samples from 16 children with suspicion of gastrointestinal infection treated at the same hospital during 2015 all tested negative with qPCR (data not shown).

The virus growing in the initial Caco-2 cell culture was therefore MRV-3 and not an enterovirus as suggested by the serotyping assay. In fact, the manufacturer’s datasheet for the reagent used states that there is potential for cross-reaction with *Hepatitis A*, *Reovirus* 3, and some *Rhinovirus* and *Astrovirus* strains. The latter highlights the genuine difficulty in accurate detection of highly diverse virus families with numerous genotypes such as *Enteroviruses* where typing by specific PCR can be challenging due to the high susceptibility to recombination and the emergence of novel strains [15]. Notably, CV-A was detected in stool suspensions but not in cell culture supernatants. While cell culturing was critical for MRV-3 detection, CV-A, in line with a general difficulty to culture coxsackieviruses [16], did not infect Caco-2 cells. The reason that MRV-3 was not detected by metagenomic sequencing in the original stool suspension, but only after amplification in cell culture, is likely because it was at levels too low to be detected with the applied depth of sequencing.

In summary, this case highlights the complexity of infections in immunocompromised hosts and reveals limitations of routine diagnostic methods. A combination of traditional cell culture, metagenomic sequencing, verification by specific PCRs and serology proved key to detect a persistent co-infection with three clinically relevant viruses. While the presence of these viruses in stool specimen suggests a link with the observed pathogenesis, it cannot be defined to what extent each of the viruses contributed to the initial flu-like symptoms and prolonged diarrhea. Based on the timing of virus detection, the earlier detected CV-A and HAdV-C are more likely to be involved in the child’s condition than the later emerging MRV-3.

## Additional file

Additional file 1: Portable Document Format. **Table S1.** Routine screening results for Enterovirus. **Figures S1 to S9.** Phylogenetic analyses of all Mammalian orthoreovirus segments isolated in this study. **Figure S10.** Coxsackievirus BLAST genotyping. **Figure S11.** Coxsackievirus Bootscan Analysis. Supplementary Methods. Supplementary References. (PDF 456 kb)

## Abbreviations

CPE: Cytopathic effect
CV-A: Coxsackievirus A
HAdV-C: Human adenovirus C
MRV: Mammalian orthoreovirus
PCR: Polymerase chain reaction
PID: Primary immunodeficiency
