# Supplementary material for: Persistent mammalian orthoreovirus, coxsackievirus and adenovirus co-infection in a child with a primary immunodeficiency detected by metagenomic sequencing: a case report
Source: BMC Infect Dis. 2018 Jan 11;18:33. doi: 10.1186/s12879-018-2946-7 (PMC5765704; doi:10.1186/s12879-018-2946-7)
Supplement: Additional file 1: — Portable Document Format. Table S1. Routine screening results for Enterovirus. Figures S1 to S9. Phylogenetic analyses of all Mammalian orthoreovirus segments isolated in this study. Figure S10. Coxsackievirus BLAST genotyping. Figure S11. Coxsackievirus Bootscan Analysis. Supplementary Methods. Supplementary References. (PDF 456 kb) [file 12879_2018_2946_MOESM1_ESM.pdf]

Supplementary Table

Table S1: Routine screening results for Enterovirus

Date	Sample	Enterovirus PCR	Cell culture (CPE)
02.01.2015	stool	nd[1]	negative[2]
30.04.2015	stool	**positive**	nd
26.05.2015	stool	**positive**	nd
30.06.2015	stool	**positive**	nd
14.07.2015	stool	**positive**	nd
10.09.2015	stool	**positive**	nd
03.11.2015	stool	**positive**	**positive**[3]

[1]not done
[2]tested on MDCK, Vero, A549 and MRC-5 cells
[3]positive on Caco-2 cells, also tested on Vero, A549 and MRC-5 cells

Supplementary Figures

Figure S1 to S9

Phylogenetic analyses of all Mammalian orthoreovirus segments isolated in this study (circle, mew716_MRV-3) reveal a close relationship with previously described isolates identified in a child in Slovenia (triangle, KF154724 - KF154733), a bat in Germany (JQ412755 - JQ412764), and pigs in Italy (KX343200 - KX343209). Phylogenetic trees were constructed in MEGA7 [17] using the Maximum Likelihood method based on the Kimura 2-parameter model. Bootstrap values from 1000 tries are shown. MRV-type and host species are depicted if available.

Figure S1: MRV Segment L1

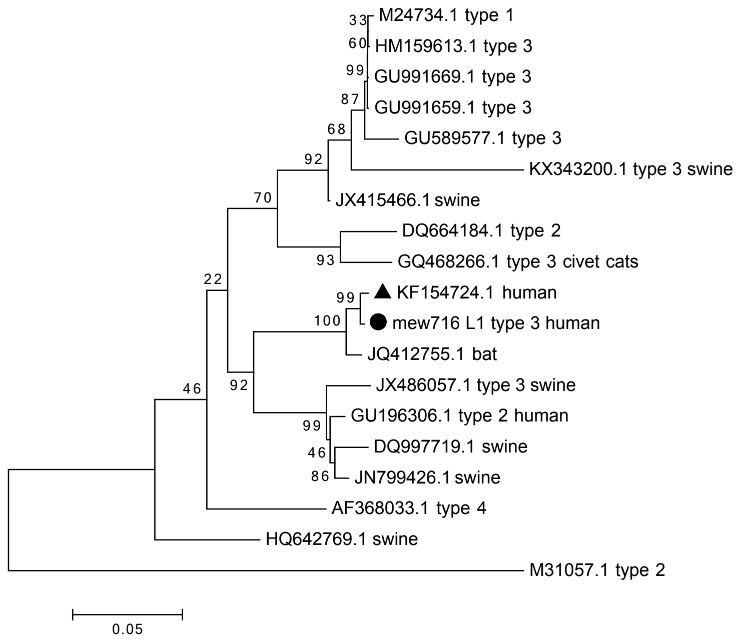

Figure S2: MRV Segment L2

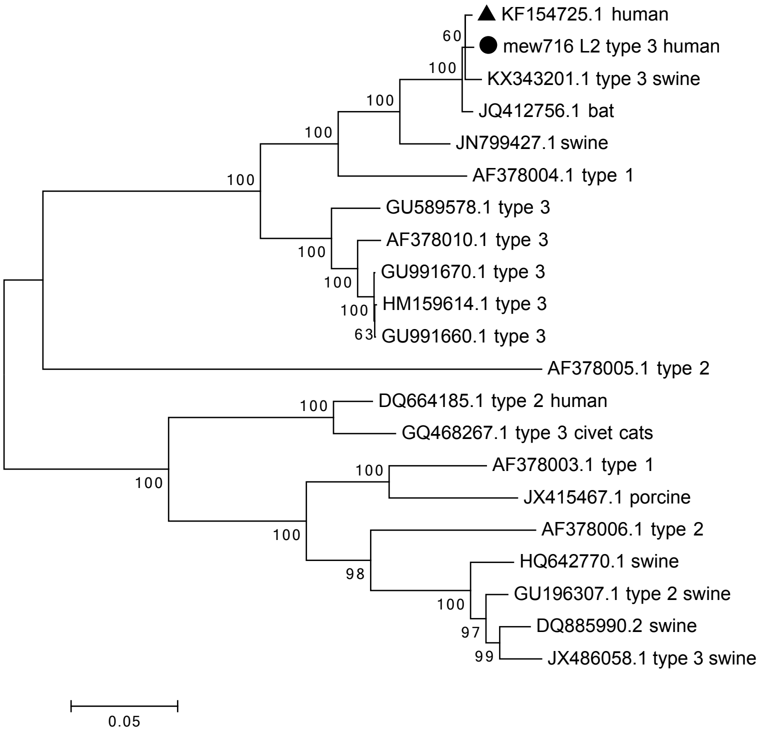

Figure S3: MRV Segment L3

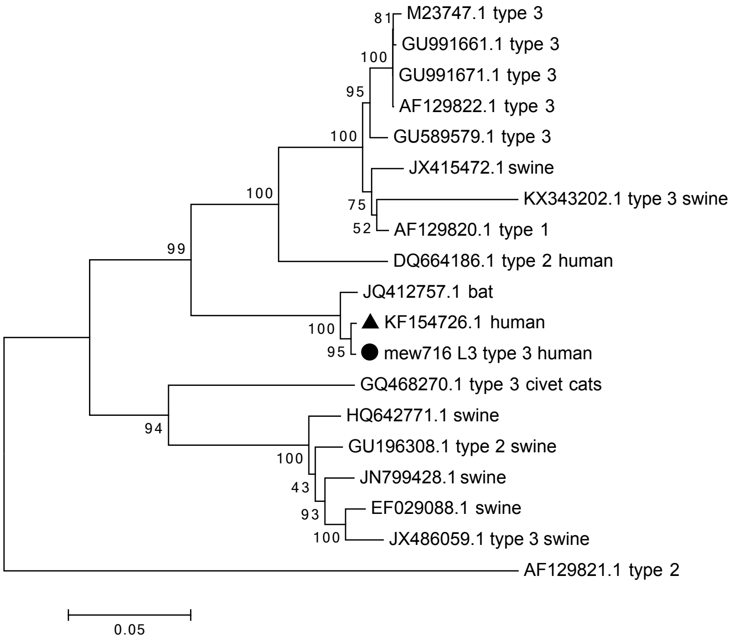

Figure S4: MRV Segment M1

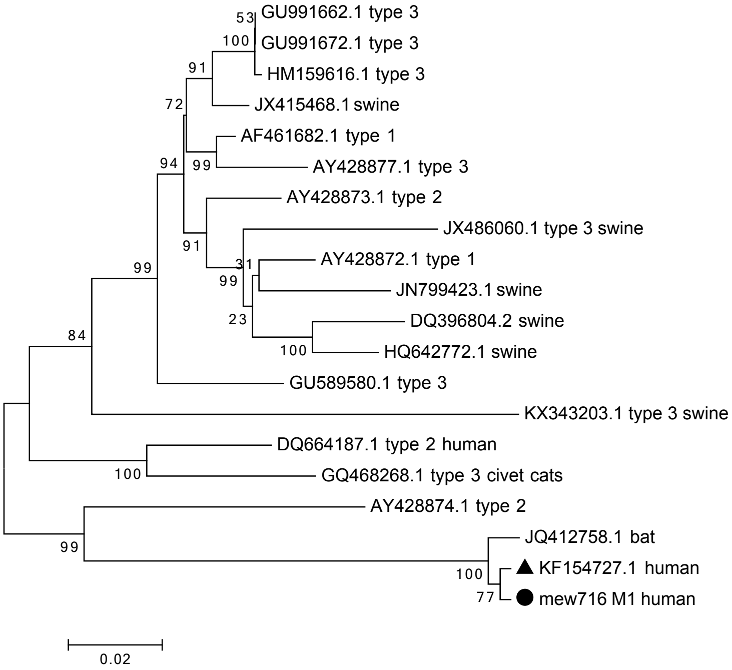

Figure S5: MRV Segment M2

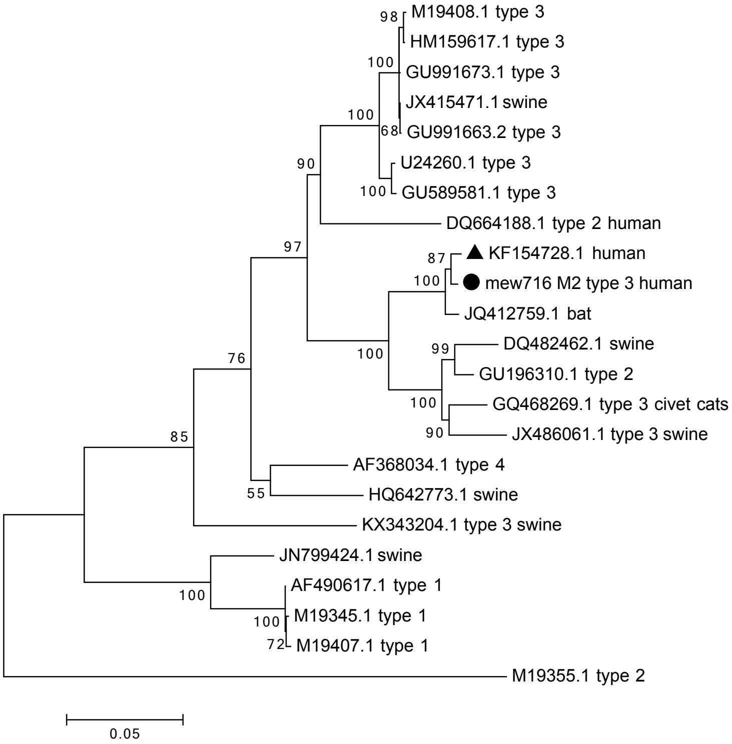

Figure S6: MRV Segment M3

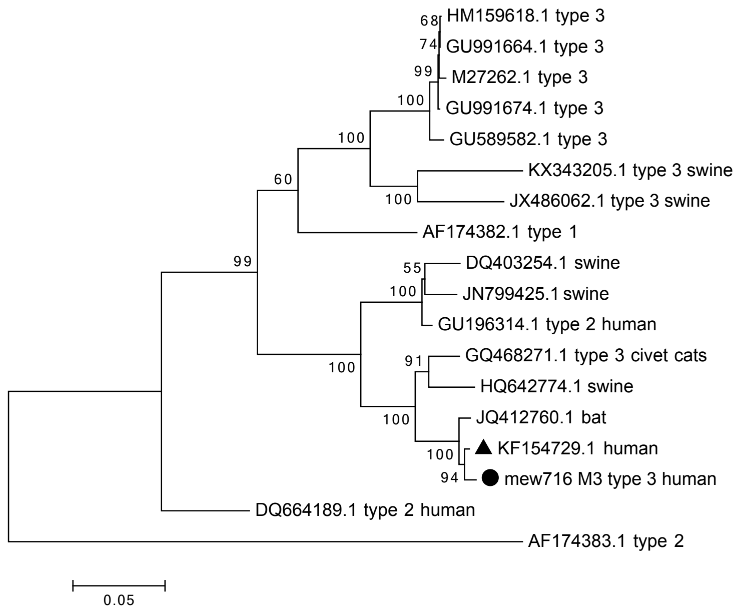

Figure S7: MRV Segment S2

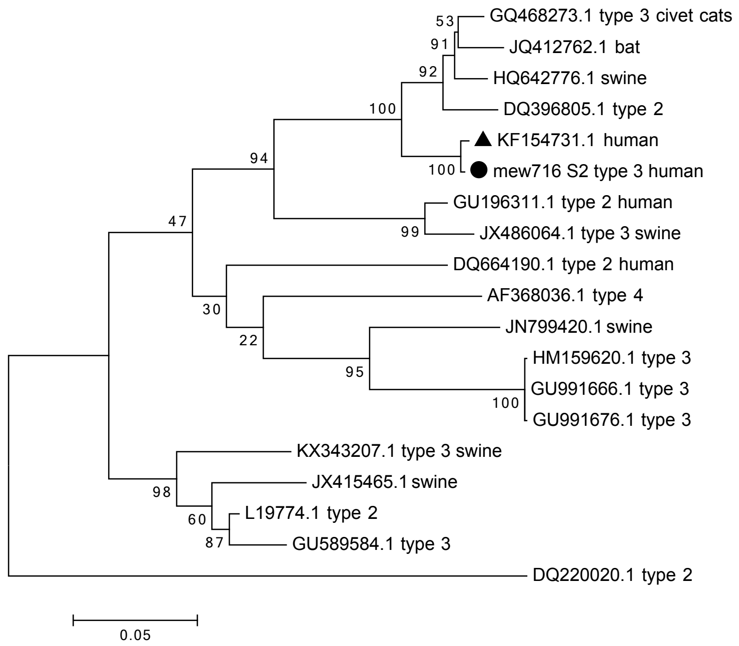

Figure S8: MRV Segment S3

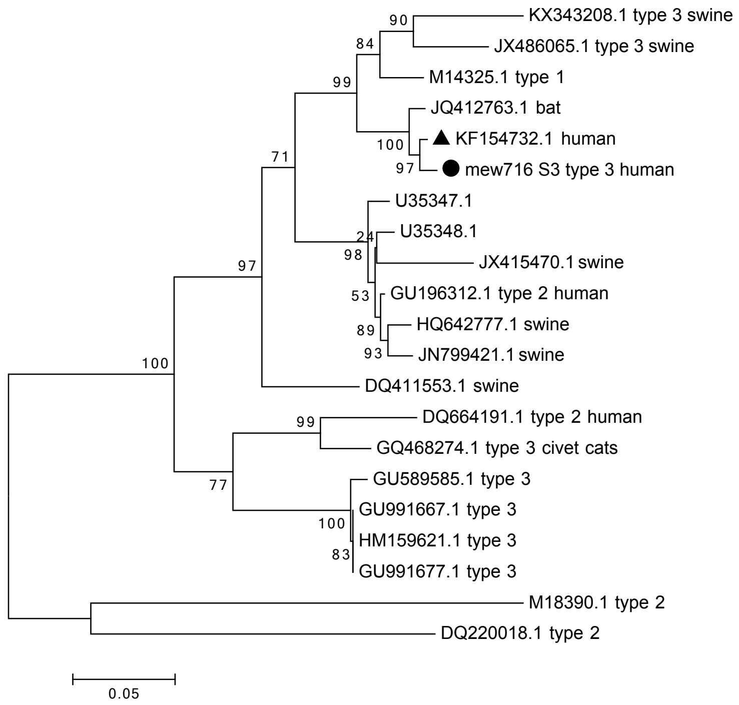

Figure S9: MRV Segment S4

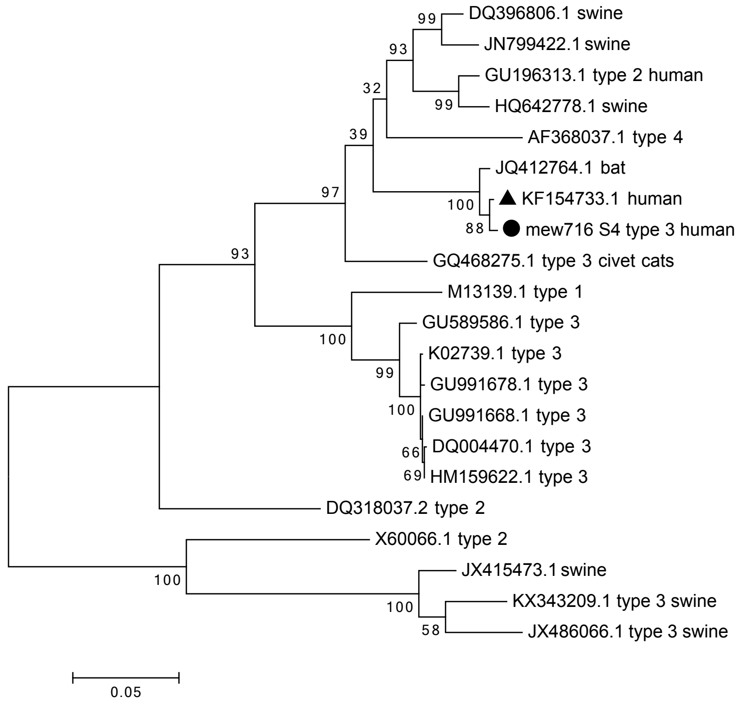

Figure S10: Coxsackievirus BLAST genotyping

Genotyping of CV-A isolate identified in this study was performed using the NCBI BLAST Genotyping tool (www.ncbi.nlm.nih.gov/projects/genotyping/formpagex.cgi) using a window size of 300, an increment size of 100 and a similarity threshold of 30%. CV-A19 genotypes are shown with a solid line, CV-A22 genotypes with a dashed line. CV-A19 strain OC01017025e (AB828290) and CV-A22 strain 438913 (JN542510) are shown in red and purple, respectively.

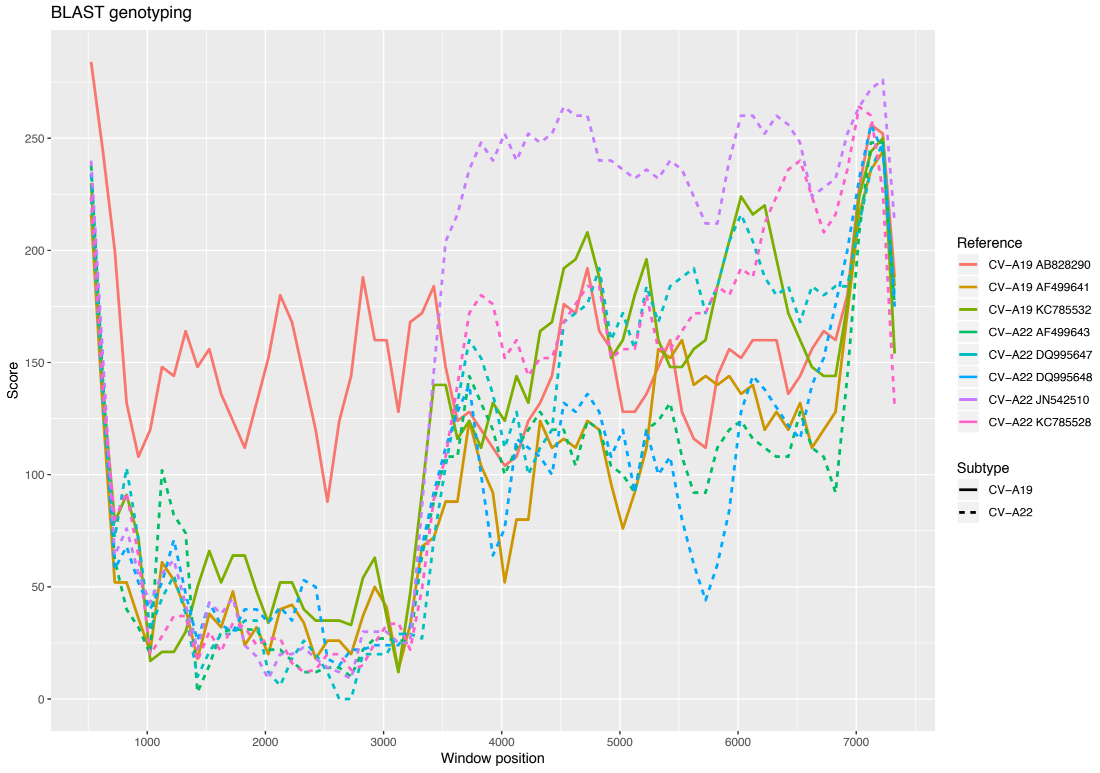

Figure S11: Coxsackievirus Bootscan Analysis

Bootscan analysis of CV-A isolate identified in this study was performed using the manual bootscan method within the RDP4 program [18, 19]. CVA19 genotypes are shown with a solid line, CVA22 genotypes with a dashed line. CV19 strain OC01017025e (AB828290) and CV22 strain 438913 (JN542510) are shown in red and purple, respectively. The bootstrap cut-off is show by a dotted horizontal line.

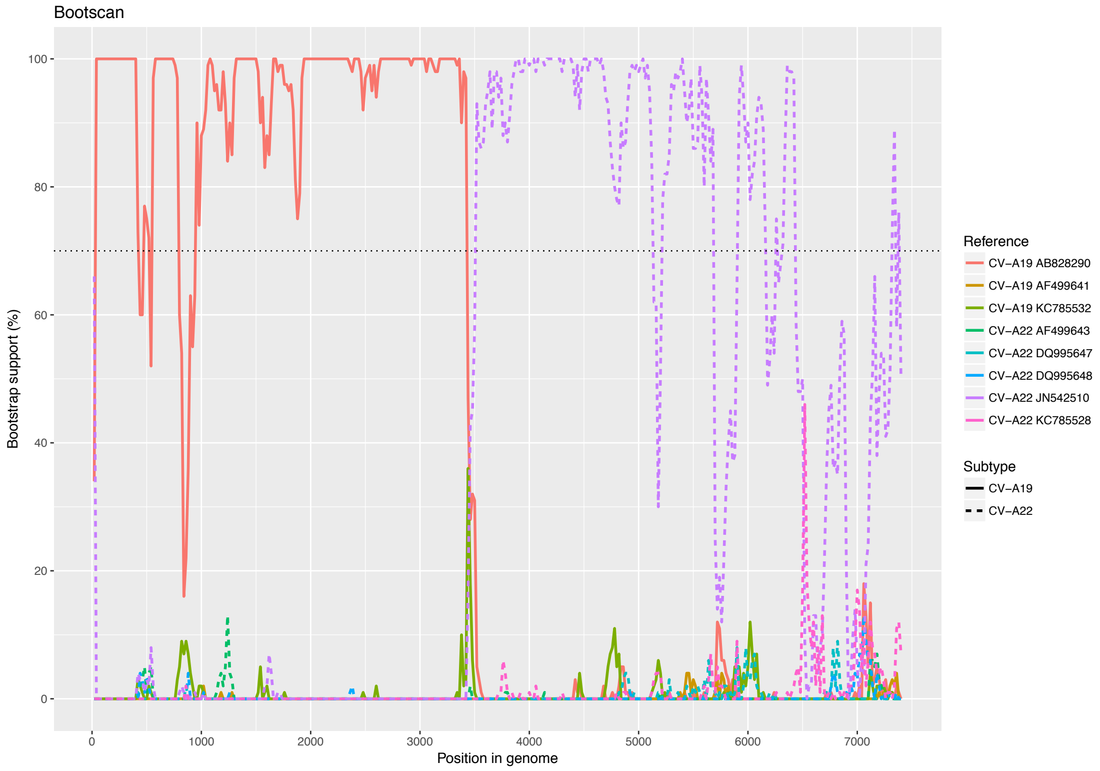

Methods

Cell culture

Caco-2 cells (epithelial colorectal adenocarcinoma, ECACC 86010201, LOT09C004, Salisbury, UK) were cultivated in M199 medium (ICN Nutritional Biochemicals, Cleveland, OH) containing 10% heat inactivated fetal calf serum (Chemie Brunschwig, Switzerland), glutamine (ICN Nutritional Biochemicals, Cleveland, OH), sodium bicarbonate (Gibco, Carlsbad, CA) and a mixture of penicillin/streptomycin (Gibco, Carlsbad, CA). Confluent cells in flat-sided culture tubes were used for the inoculation of specimens. Stool samples were suspended in PBS (10% v/v), frozen at -80°C overnight, unfrozen in lukewarm water and centrifuged at 3000 g for 30 min. The supernatant was then removed and penicillin, streptomycin, gentamycin (Biochrom GmbH, Berlin, Germany) and amphotericin B/fungizone (Gibco, Carlsbad, CA) were added. The suspension was incubated for 10 min at 4°C. Culture tubes were inoculated with 200 μl of the suspension in 2 ml culture medium and then incubated at 37°C in a CO_2 incubator. Cultures were screened periodically for cytopathic changes for 14 days and compared with a non-infected cell-culture control. If rounding up of cells started to appear, they were scraped off and used to prepare cytospin slides for immunofluorescence staining with a pool of anti-enterovirus monoclonal antibodies (pan-enterovirus reagent, Light-Diagnostics/Merck).

Routine Enterovirus typing

After drying, fixation and washing of the slides with 1% milk powder solution, cells were incubated with monoclonal antibodies directed against enteroviruses. Entrovirus typing is a stepwise procedure and included the following monoclonal antibody pools (blends): Pan Entero blend (Light Diagnostics Pan-Enterovirus Reagent, Merck, Darmstadt, Germany), Polio blend Entero blend, Echo blend and Coxsackie blend (all from Light Diagnostics, Merck). Of note, the antigen specificity of the antibodies in the Pan Entero blend is not

specified and potential cross-reaction with hepatitis A, reovirus 3, and some rhinovirus and astrovirus strains is indicated in the data sheet.

Routine Enterovirus and Adenovirus PCR

Extracted nucleic acids (5 µl) were used for Enterovirus specific PCR [20]. Primers and probes were synthesized by Microsynth AG (Balgach, Switzerland) and Applied Biosystems (Life Technologies), respectively. Primer concentrations were 1 µM each, probe concentrations was 0.4 µM in a reaction volume of 50 µl. Amplification and detection were performed with the QuantiTect Probe RT-PCR Kit (Qiagen AG, Hombrechtikon, Switzerland) on an ABI 7300 instrument (Applied Biosystems/Life Technologies) with cycling conditions 30 min 48°C, 10 min 95°C followed by 50 cycles 15 sec at 95°C, 1 min at 60°C.

Adenovirus PCR was performed with probe and primers described before [21]. Reaction was performed on a ViiA7 Real-Time PCR System (Life Technologies Europe, Zug, Switzerland) with the TaqMan RNA-to-Ct 1-Step Kit, with 10 µl master mix, 1 µl template, 10 µM primers and probes in a total volume of 20 µl. Each PCR was run in duplicates with the following cycling conditions: 10 min 95 °C followed by 50 cycles 15 sec at 95 °C, 1 min at 60 °C.

Specific Mammalian Orthoreovirus and Coxsackievirus PCR

Orthoreovirus primer and probe were: TCACAATCCCCCATCACTATT, CATCCATTTCTGCCAGTTCTT, and FAM-ATAAATGGTATCAGGGCGCGCAGGAG-TAMRA. Coxsackievirus primer and probe were: TGCCAGTCCAAGGATTTCAA, GCACCAGCATCCACCTG, and FAM-TGGGAATTGCATCGGCTTACTCGCACTTCT-TAMRA. PCRs were performed on a QuantStudio 7 (Thermo Fisher Scientific) with the TaqMan RNA-to-Ct 1-Step Kit (Thermo Fisher Scientific), 1 µl template, 0.5 µM primers and 0.4 µM probe in an end volume of 20 µl. Each PCR was run in duplicates with the following cycling conditions: 50 °C for 2 min; 95 °C for 10 min; 45 cycles 95 °C for 15 sec, 55 °C for 1 min.

Metagenomic Sequencing

Cell-culture supernatant was centrifuged and filtered (0.4 µm, TPP, Trasadingen, Switzerland) and used for metagenomic sequencing. Metagenomic sequencing was performed as describe previously [4, 22]. Briefly, after virus enrichment and extraction, RNA and DNA were amplified in two separate workflows. A random octamer primer with an anchor sequence was used for reverse transcription (SuperScript III, Invitrogen) and one round of second-strand synthesis (DNA Polymerase I, Large Klenow Fragment, NEB). Both workflows were further amplified using the anchor part alone and pooled in equal amounts for library construction with NexteraXT (Illumina), prior to sequencing for 150 bp on an Illumina MiSeq system. Reads were analysed using the "VirMet" bioinformatics pipeline (https://github.com/ozagordi/ VirMet/releases/tag/v0.3.3). Briefly, reads were quality-filtered by removing low quality bases (average PHRED score below 20), reads shorter than 75 bp and reads with low entropy (i.e., consisting mainly of repeats). Read passing quality filters were cleaned from non-viral reads by aligning with STAR [23, 24] against, in this order, human, bacterial, bovine, and canine genomes. Reads not matching any of the above genomes were aligned with BLAST [25] against an in-house viral database that contains approximately 46,000 different virus sequences. For each sequencing read that passed the quality filter, the BLAST hit with lowest e value was reported, given the identity was higher than 75%. Reads which did not match genomes used in the cleaning step and did not match viral genomes included in the database were reported as of unknown origin [4, 22].

Full-length coxsackie virus sequencing and recombination analysis

In addition to metagenomic sequencing, the full Coxsackievirus VP2, VP3 and VP1 region was amplified with serotype consensus primers [26] and the amplicon sequenced using NexteraXT protocols. The full genome was constructed from all available sequencing information by doing iterative alignments with bwa.

Detection of MRV specific antibodies by immunofluorescence

MRV-3 positive cell cultures were fixed on slides and incubated with different patient and control sera. A secondary FITC-anti-human IgG antibody (Meridian Biosciences) was used for the detection of human MRV-specific IgG antibodies. A monoclonal antibody against Reovirus Type 3 (Sigma-Aldrich/Merck) was used as positive control.

Supplementary References

17. Kumar S, Stecher G, Tamura K. MEGA7: Molecular Evolutionary Genetics Analysis Version 7.0 for Bigger Datasets. Molecular biology and evolution. 2016;33(7):1870-4.
18. Martin DP, Lemey P, Lott M, Moulton V, POSADA D, Lefeuvre P. RDP3: a flexible and fast computer program for analyzing recombination. Bioinformatics. 2010;26(19):2462-3.
19. Martin DP, Posada D, Crandall KA, Williamson C. A modified bootscan algorithm for automated identification of recombinant sequences and recombination breakpoints. AIDS Res. 2005;21(1):98-102.
20. Tapparel C, Cordey S, Van Belle S, Turin L, Lee WM, Regamey N, Meylan P, Muhlemann K, Gobbini F, Kaiser L. New molecular detection tools adapted to emerging rhinoviruses and enteroviruses. Journal of Clinical Microbiology. 2009;47(6):1742-9.
21. Heim A, Ebnet C, Harste G, Pring-Akerblom P. Rapid and quantitative detection of human adenovirus DNA by real-time PCR. J Med Virol. 2003;70(2):228-39.
22. Lewandowska DW, Zagordi O, Geissberger FD, Kufner V, Schmutz S, Böni J, Metzner KJ, Trkola A, Huber M. Optimization and validation of sample preparation for metagenomic sequencing of viruses in clinical samples. Microbiome. 2017;5(1):115.
23. Dobin A, Gingeras TR. Mapping RNA-seq Reads with STAR. Curr Protoc Bioinformatics. 2015;51(11.4.1-.4.9.
24. Dobin A, Davis CA, Schlesinger F, Drenkow J, Zaleski C, Jha S, Batut P, Chaisson M, Gingeras TR. STAR: ultrafast universal RNA-seq aligner. Bioinformatics. 2013;29(1):15-21.
25. Camacho C, Coulouris G, Avagyan V, Ma N, Papadopoulos J, Bealer K, Madden TL. BLAST+: architecture and applications. BMC bioinformatics. 2009;10(421.
26. Tokarz R, Haq S, Sameroff S, Howie SRC, Lipkin WI. Genomic analysis of coxsackieviruses A1, A19, A22, enteroviruses 113 and 104: viruses representing two clades with distinct tropism within enterovirus C. J Gen Virol. 2013;94(Pt 9):1995-2004.
